# Prognosis analysis of sacrococcygeal teratoma—compared with patient size

**DOI:** 10.3389/fped.2025.1513825

**Published:** 2025-03-24

**Authors:** Jueun Park, Suhyeon Ha, Hyunhee Kwon, Seong Chul Kim, Jung-Man Namgoong, Dae Yeon Kim

**Affiliations:** Division of Pediatric Surgery, Asan Medical Center Children’s Hospital, Asan Medical Center, University of Ulsan College of Medicine, Seoul, Republic of Korea

**Keywords:** sacrococcygeal teratoma, prognosis, surgical complication, pediatric surgery, pediatric oncology

## Abstract

**Introduction:**

Advancements in prenatal care have underscored the importance of understanding postnatal outcomes and prognosis in sacrococcygeal teratoma (SCT). However, giant SCTs continue to pose surgical challenges and are associated with increased morbidity and mortality. A clear, objective threshold for defining a “large” tumor relative to the patient's size remains undefined. This study aimed to establish objective tumor size indicators for predicting surgical outcomes and prognosis.

**Methods:**

Data from 97 patients diagnosed with and surgically treated for SCT at Asan Medical Center from 2000 to 2021 was retrospectively reviewed. The tumor volume/birthweight ratio (VWR) and tumor length/height at birth ratio (LHR) were measured. Surgical outcomes and prognosis were evaluated based on these measures, including surgical complications, concomitant surgeries, long-term complications, and recurrence.

**Results:**

After surgery, 38 patients experienced short-term complications, 18 required additional operations, 16 developed long-term complications, and 14 experienced tumor recurrence. Both VWR and LHR correlated with short and long-term complications, additional surgeries, and recurrence.

**Discussion:**

The small patient population limited the determination of precise cut-off values; however, a significant difference was observed between groups stratified by the most predictive cut-off values. Both objective tumor size indicators were significantly associated with prognosis and surgical outcomes. Notably, both indicators exhibited comparable predictive capabilities without discrepancies.

## 1 Introduction

Sacrococcygeal teratoma (SCT) is one of the most prevalent congenital neoplasms, occurring in approximately 1 in 35,000 to 40,000 live births (1). When detected via routine prenatal ultrasonography *in utero*, smaller SCTs have a survival rate exceeding 90% (2–5). However, large, solid tumors with dense vascularity and rapid growth are associated with poor fetal outcomes (6–8). There was a 50% mortality with tumors exciding 10 cm in highly vascular or fast growing, contrasting starkly with cases lacking these characteristics or predominantly comprising cystic formations, where no fatalities were recorded (9). Moreover, mortality increases to nearly 100% in fetuses with hydrops or placentomegaly (2–5). Even if a fetus with SCT is delivered safely and survived, excision of a large vascular SCT in a newborn poses life-threatening risks, including hemolysis, rupture, or excessive bleeding (10). Additionally, the anatomical location of the tumor may increase the risk of injury to the pelvic viscera, including injury to the urinary collection system and lower gastrointestinal tract (6, 10, 11).

Altman et al. (12) introduced a four-stage classification system for SCTs based on anatomical location (13). Additionally, Altman categorized SCTs as small (2–5- cm in diameter), moderate (5–10- cm in diameter) and large (>10- cm in diameter) (12). This classification provides insight into the potential ease of surgical resection. However, no definitive objective indicator currently exists for assessing tumor size relative to patient size. Studies have shown that a tumor-fetus ratio (TFR) greater than 0.12 before 24 weeks of gestation is associated with poor fetal prognosis (14, 15). However, these studies primarily focused on fetal outcomes, such as fetal demise, and did not examine the impact of tumor size on postnatal prognosis and surgical management.

Thus, this study evaluated the postnatal prognosis of SCT based on tumor size relative to patient size. Furthermore, if possible, we aimed to establish a cut-off value for defining “large SCT” in relation to prognosis.

To achieve this, we retrospectively analysed treatment outcomes and prognoses in patients with SCT who underwent surgical treatment at a single center, considering tumor size relative to patients’ size.

## 2 Patients and methods

### 2.1 Patient population

Data were collected from patients with SCT who underwent surgery at Seoul Asan Medical Center between January 2000 and December 2021. Patients without radiological test results for tumor volume or length measurement were excluded. The Institutional Review Board of Asan Medical Center approved this study (IRB no. 2022-0487).

### 2.2 Data collection

Patient data were categorized based on prenatal factors (i.e., polyhydramnios, associated anomalies, hydrops, need for fetal intervention, gestational age at birth, and mode of delivery) and tumor characteristics (i.e., radiological volume, radiological length, pathological weight, pathological length, Altman type, and histopathology). Surgical treatment outcomes were assessed in terms of perioperative complications, late complications, recurrence and additional operations. An additional operation was defined as a subsequent procedure required to address a complication or anomaly. Recurrence was identified based on the detection of a new lesion on abdominopelvic magnetic resonance imaging (MRI), abdominopelvic computed tomography (CT), or ultrasonography (US).

### 2.3 Relative size indicators

Relative tumor size was assessed using two indicators: one based on volume (tumor volume to net birth weight ratio) and one based on length (radiological tumor length to birth height ratio). The volume-weight ratio (VWR) was calculated by dividing the tumor volume by net birth weight. Tumor volume was determined using in-house computer-aided volumetry software based on MRI or CT images. Volumetry was performed using a manual planimetry technique on axial slices, where cross-sectional slices were manually traced. Tumor volume was then calculated by multiplying the traced areas by the slice thickness and summing the resulting slice volumes. Net birth weight was obtained by subtracting tumor volume from birth weight.

The length-height ratio (LHR) was calculated by dividing the longest tumor length by birth height. Tumor length was determined through radiological evaluations, including CT, MRI, or US. Patients who underwent only US and whose tumor length could not be measured or whose Altman type could not be determined were excluded from the respective analyses.

### 2.4 Data analysis

Statistical analyses were performed using SAS version 9.4 (SAS Institute Inc., Cary, NC, USA). Each indicator and prognostic outcome was analyzed using logistic regression analysis. For the “recurrence” of the outcome, data were analyzed by Cox regression. Receiver operating characteristic curve (ROC) analysis was used to select the cut-off values of VWR and LHR based on the maximum Youden index value—the optimal cut-off threshold that maximizes both sensitivity and specificity. An ROC curve graph was made using MedCalc, and citation was made for the patients' characteristics, tumor characteristics and prognostic factor analysis. Continuous values were tested using the Mann–Whitney *U*-test, and categorical values were tested using the chi-square test for Fisher's exact test. *P*-value <0.05 were used to denote statistical significance.

## 3 Results

A total of 97 patients underwent surgery for SCT between January 2000 and December 2021. Ten patients did not undergo CT or MRI before surgery because imaging was not performed in time due to scheduling constraints or because the patient's condition, such as a ruptured tumor requiring emergency surgery, prevented imaging. Instead, US was performed. However, in seven patients, measuring the tumor length using sonography was not possible because the tumor was too large, surpassing the size of the ultrasound probe, and because of the retrospective nature of data collection. Therefore, 87 patients were included in the VWR analysis, and 90 patients were included in the LHR analysis.

### 3.1 Need for additional operation

Eighteen additional surgeries were performed including mass re-excision (14 cases), stoma formation (3 cases), anoplasty (2 cases), and bleeding control (1 case). The mean VWR in the additional surgery group was 28.75 ± 34.46(13.3), while in the single-surgery group, it was 8.84 ± 16.91(2.76). The mean LHR values were 27.18 ± 14.12(25.43) and 17.25 ± 10.95(14.73) in the additional and single-surgery groups, respectively.

**Figure 1:**
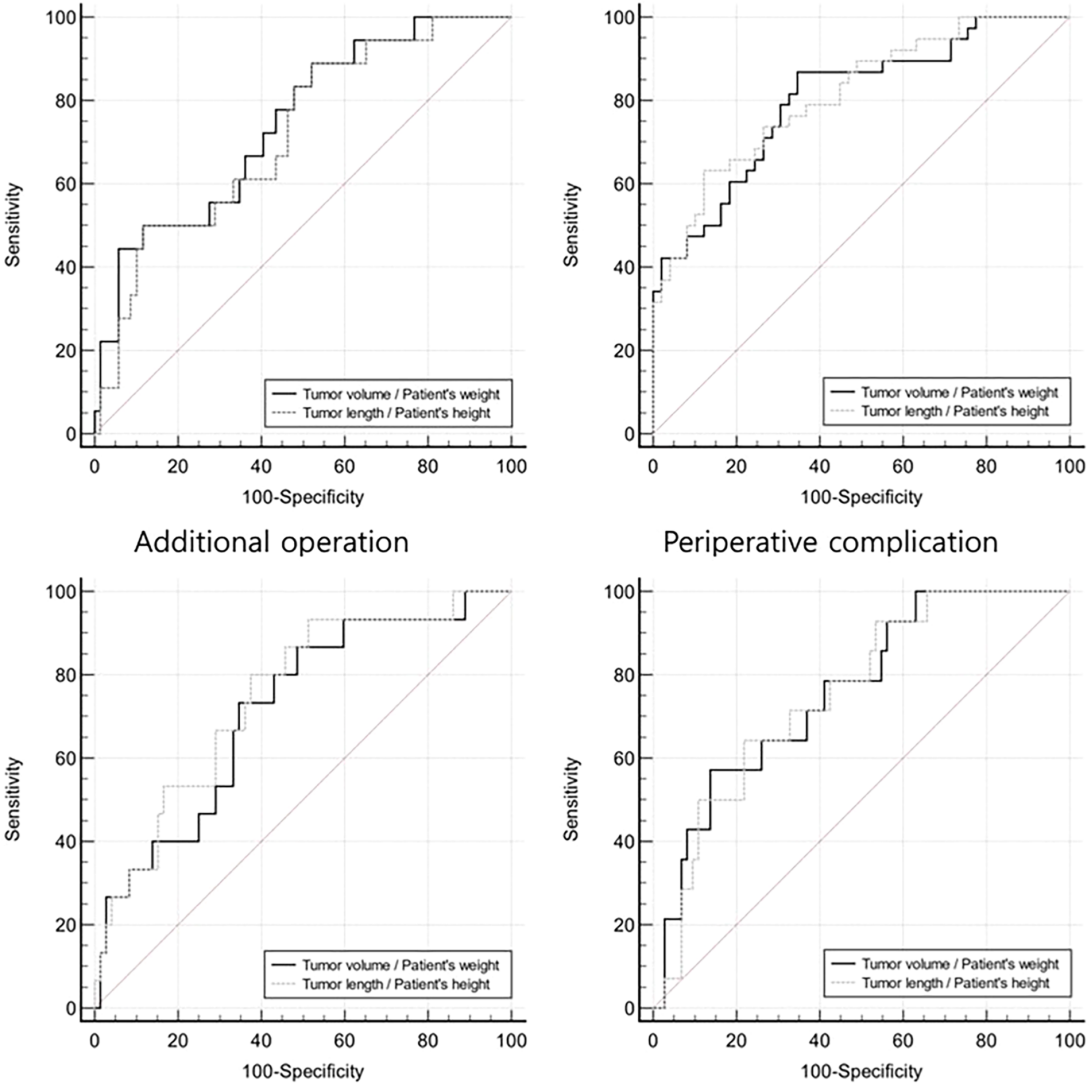
Receiver operating characteristic curve for four outcomes (additional operation, perioperative complication, late complication, and recurrence), illustrating the sensitivity and specificity of VWR and LHR.

Both VWR and LHR were associated with the likelihood of requiring additional surgery, with odds ratios of 1.033(*p* = 0.0088) and 1.062 (*p* = 0.0047), respectively. Figure 1 shows the ROC curve for additional surgeries (top left). The estimated cut-off value for VWR was 27.4, with 44% sensitivity and 94% specificity, while the estimated cut-off value for LHR was 28.2, with 50% sensitivity and 88% specificity. The area under the curve (AUC) values for VWR and LHR were 0.744 and 0.719, respectively. Both indicators were significantly associated with the additional operation, with *p*-values of 0.0002 and 0.001, respectively. No statistically significant difference was observed between the two indicators (*p* = 0.0599).

To differentiate between reoperation and additional surgery for complication, the additional operation group was divided into the additional mass excision group and the additional anorectal operation group. VWR and LHR were associated with additional mass excision, with odds ratios of 1.024 (*p* = 0.0305) and 1.054 (*p* = 0.0148), respectively. However, no association was observed with additional anorectal surgeries, with odds ratios of 1.023 (*p* = 0.0841) for VWR and 1.046 (*p* = 0.1795) for LHR.

### 3.2 Perioperative complications

Thirty-eight patients experienced perioperative complications, including intraoperative transfusion (27 cases), postoperative ventilator use for more than 24 h (27 cases), hypoxic damage (1 case), and urinary tract injury (3 cases).

The mean VWR values for the perioperative complication and no-complication group were 24.37 ± 30.66(8.89) and 4.11 ± 5.99 (1.62), respectively. The mean LHR values were 26.94 ± 13.67 (23.41) and 13.61 ± 7.01 (13.07), respectively. Both VWR and LHR were associated with perioperative complications, with odds ratios of 1.105 (*p* = 0.0008) and 1.149 (*p* < 0.0001), respectively.

Figure 1 (top right) presents the ROC curve for perioperative complications. The cut-off values were 2.7 for VWR (87% sensitivity, 65% specificity) and 20.4 for LHR (63% sensitivity, 88% specificity). The AUC values for VWR and LHR were 0.803 and 0.812, respectively. Both indicators were significantly associated with perioperative complications (*p* < 0.0001). No statistically significant difference was observed between the indicators (*p* = 0.6081).

### 3.3 Late complications

Late complications were assessed during routine outpatient follow-ups. Nine patients experienced defecation problems, and seven had voiding problems, requiring continuous outpatient visits.

The mean VWR values for the late complication and no-complication groups were 26.84 ± 34.64 (6.79) and 10.07 ± 18.77 (2.8), respectively. The mean LHR values were 28.87 ± 14.86 (25.53) and 17.31 ± 10.76 (14.74), respectively.

Both VWR and LHR were associated with late complications, with odds ratios of 1.024 (*p* = 0.0263) and 1.07 (*p* = 0.0027), respectively. Figure 1 (bottom left) shows the ROC curve for late complications. The cut-off values were 4.8 for VWR (73% sensitivity, 65% specificity) and 17.1 for LHR (80% sensitivity, 62.5% specificity). The AUC values were 0.716 and 0.747, respectively. Both indicators were significantly associated with late complications (*p* = 0.0027 and *p* = 0.0003). No statistically significant difference was observed between the indicators (*p* = 0.0875).

### 3.4 Recurrence

Recurrence was identified based on new lesions observed on abdominopelvic US, CT, or MRI. Fourteen patients experienced recurrence.

The mean VWR values for the recurrence and no-recurrence groups were 26.05 ± 25.7 (19.54) and 10.45 ± 21.68 (3.16), respectively. The mean LHR values were 27.54 ± 11.75 (26.92) and 17.71 ± 11.77 (14.92), respectively.

Both indicators were significantly associated with recurrence, with hazard ratios of 1.02 (*p* = 0.0076) for VWR and 1.049 (*p* = 0.0038) for LHR. ROC curve analysis showed that both indicators were associated with recurrence, with AUC values of 0.760 (*p* = 0.0001) and 0.753 (*p* = 0.0001), respectively. No significant difference was observed between the indicators (*p* = 0.6486). Figure 1 (bottom right) shows the ROC curve for recurrence, with cut-off values of 18.3 for VWR (57% sensitivity, 86% specificity) and 22.4 for LHR (64% sensitivity, 78% specificity).

### 3.5 Cut-off value

Since the ROC curve analysis yielded different cut-off values for each outcome, the most predictive cut-off values were selected: 18.5 for VWR and 20.5 for LHR, based on sensitivity and specificity.

Using these cut-off values, the patients were divided into two groups, and their baseline characteristics are presented in Table 1. Continuous variables are reported as medians (Q1-Q3) and were analyzed using the Mann–Whitney *U*-test, wihle categorical variables are presented as numbers (percentages) and were analyzed using the chi-square test or Fisher's exact test. Significant differences were observed in all factors between the VWR >18.5 and VWR ≤ 18.5 groups. The high VWR group had a greater prevalence of preterm birth, low birth weight, cesarean section, associated anomalies, polyhydramnios, hydrops and fetal intervention. In the LHR groups, birth weight and hydrops did not significantly differ between the groups.

**Table 1:** Patients’ characteristics divided by cut off value.

	VWR > 18.5 (*n* = 18)	VWR ≤ 18.5 (*n* = 69)	*p*-value	LHR > 20.5 (*n* = 31)	LHR ≤ 20.5 (*n* = 59)	*p*-value
Gestational age			<.0001			<0.0001
≥37 weeks	4 (22.22)	59 (85.51)		12 (38.71)	53 (89.83)	
<37 weeks	14 (77.78)	10 (14.49)		19 (61.29)	6 (10.17)	
Birth weight (g)			0.0152			0.0828
≥2,500	13 (72.22)	64 (92.75)		25 (80.65)	54 (91.53)	
1,500–2,499	5 (27.78)	3 (4.35)		6 (19.35)	3 (5.08)	
1,000–1,499	0 (0.00)	1 (1.45)		0 (0.00)	1 (1.69)	
<1,000	0 (0.00)	1 (1.45)		0 (0.00)	1 (1.69)	
Mode of delivery			0.0022			0.0055
Vaginal delivery	0 (0.00)	24 (34.78)		3 (9.68)	22 (37.29)	
Caesarian section	18 (100)	45 (65.22)		28 (90.32)	37 (62.71)	
Associated anomaly	9 (50)	13 (18.84)	0.0128	13 (41.94)	9 (15.25)	0.0051
Polyhydramnios	12 (66.67)	3 (4.35)	<.0001	14 (45.16)	1 (1.69)	<0.0001
Hydrops	2 (11.11)	0 (0.00)	0.0409	2 (6.45)	0 (0.00)	0.1161
Fetal intervention	14 (77.78)	12 (17.39)	<.0001	17 (54.84)	9 (15.25)	0.0001

Continous values are presented by median (Q1–Q3) and tested by Mann–Whitney *U*-test, and categorical values are presented by *n* (%) and tested by chi-squared test or Fisher's exact test.

Table 2 summarizes the tumor characteristics of the two groups. No significant differences were observed in “Altman type” or “complicated tumor”. However, histopathologic biopsy results indicated a higher proportion of immature tumors in the large VWR and LHR groups (*p* < 0.001).

**Table 2:** Tumor characteristics divided by cut off value.

	VWR > 18.5 (*n* = 18)	VWR ≤ 18.5 (*n* = 69)	*p*-value	LHR > 20.5 (*n* = 31)	LHR ≤ 20.5 (*n* = 59)	*p*-value
Histopathology			<.0001			<0.0001
Mature	2 (11.11)	55 (79.71)		10 (32.26)	50 (84.75)	
Immature	16 (88.89)	14 (20.29)		21 (67.74)	9 (15.25)	
Altman type			0.0773			0.0682
1	8 (44.44)	17 (24.64)		12 (38.71)	15 (25.42)	
2	8 (44.44)	22 (31.88)		13 (41.94)	18 (30.51)	
3	2 (11.11)	22 (31.88)		6 (19.35)	18 (30.51)	
4	0 (0.00)	8 (11.59)		0 (0.00)	8 (13.56)	
Complicated tumor			0.2282			0.178
Ruptured	4 (22.22)	7 (10.14)		6 (19.35)	5 (8.47)	
Unruptured	14 (77.78)	62 (89.86)		25 (80.65)	54 (91.53)	

Continous values are presented by median (Q1–Q3) and tested by Mann–Whitney *U*-test, and categorical values are presented by *n* (%) and tested by chi-squared test or Fisher's exact test.

Outcome analysis based these cut-off values is presented in Table 3. The high VWR group was associated with poorer outcomes, although no significant differences were found for urinary tract injury, hypoxic damage, late complications, or defecation problems. Similarly the high LHR group was associated with poorer outcomes, but no significant differences were observed for urinary tract injury, hypoxic damage, or defecation problems.

**Table 3:** Outcomes divided by cut off value.

	VWR > 18.5 (*n* = 18)	VWR ≤ 18.5 (*n* = 69)	*p*-value	LHR > 20.5 (*n* = 31)	LHR ≤ 20.5 (*n* = 59)	*p*-value
Recurrence	8 (44.44)	6 (8.7)	0.0011	9 (29.03)	5 (8.47)	0.0152
Disease free date (months)	45.67 (6.53–87.5)	85.03 (31.97–127.4)	0.0181	42.77 (9.5–102.43)	90.5 (48.9–147.8)	0.004
Additional operation	9 (50)	9 (13.04)	0.0016	10 (32.26)	8 (13.56)	0.0351
Anorectal operation	1 (5.56)	3 (4.35)	1	2 (6.45)	2 (3.39)	0.6056
Mass excision	8 (44.44)	6 (8.7)	0.0011	8 (25.81)	6 (10.17)	0.0684
Perioperative complication	16 (88.89)	22 (31.88)	<.0001	24 (77.42)	14 (23.73)	<0.0001
Intraoperative transfusion	13 (72.22)	14 (20.29)	<.0001	20 (64.52)	7 (11.86)	<0.0001
Postoperative Ventilator	14 (77.78)	13 (18.84)	<.0001	19 (61.29)	8 (13.56)	<0.0001
Urinary tract injury	2 (11.11)	1 (1.4)	0.2020	2 (6.45)	1 (1.69)	0.5640
Hypoxic damage	1 (5.56)	0 (0.00)	0.2069	1 (3.23)	0 (0.00)	0.3444
Late complication	6 (33.33)	9 (13.04)	0.0737	9 (29.03)	6 (10.17)	0.0225
Defecation problems	1 (5.56)	8 (11.59)	0.6782	4 (12.9)	5 (8.47)	0.4886
Voiding problems	5 (27.78)	2 (2.9)	0.0037	6 (19.35)	1 (1.69)	0.0062
ICU stay	18.5 (10–48)	10 (6–13)	0.0031	12 (9–25)	9 (6–13)	0.0036
Hospital stay	22 (14–48)	13 (11–16)	0.0004	17 (13–34)	13 (11–16)	0.0007

Continous values are presented by median (Q1–Q3) and tested by Mann–Whitney *U*-test, and categorical values are presented by *n* (%) and tested by chi-squared test or Fisher's exact test.

Since size indicators (i.e., VWR and LHR) do not account for all outcomes, the potential influence of intrapelvic tumor extent and tumor histopathology on prognosis was evaluated. A sub-analysis was conducted based on Altman type and histopathology. Tumors were classified as extrapelvic type (type I and II) or intrapelvic type (type III and IV) for the Altman type analysis, but no significant findings were observed (Table 4-1). However, histopathologic classification revealed several notable findings (Table 4-2). Although statistical significance was not observed for some factors, the immature tumor group generally exhibited poorer outcomes, except for additional anorectal operations and late complications.

**Table 4-1:** Outcomes according to altman type.

	Altman I/II (*n* = 64)	Altman III/IV (*n* = 32)	*p*-value
Recurrence	12 (18.75)	2 (6.25)	0.1315
Disease free date (months)	78.98 (20.02–114.82)	104.62 (47.38–151.9)	0.1346
Additional op	16 (25)	3 (9.38)	0.0701
Anorectal op	4 (6.25)	1 (3.13)	0.6619
Mass excision	13 (20.31)	2 (6.25)	0.0833
Perioperative complication	29 (45.31)	11 (34.38)	0.3055
Intraop transfusion	21 (32.81)	8 (25)	0.4319
Postop Ventilator	23 (35.94)	6 (18.75)	0.0838
Urinary tract injury	2 (3.13)	1 (3.13)	1.0000
Hypoxic damage	2 (3.13)	0 (0.00)	0.5509
Postop renal failure	2 (3.13)	0 (0.00)	0.5509
Others	3 (4.69)	0 (0.00)	0.5485
Late complication	10 (15.63)	6 (18.75)	0.6985
Defecation problems	5 (7.81)	4 (12.5)	0.4755
Voiding problems	5 (7.81)	3 (9.38)	1.0000
ICU stay	10 (8–19.5)	10 (5.5–13)	0.0845
Hospital stay	14 (11–21)	13 (11–18)	0.3016

Continous values are presented by median (Q1–Q3) and tested by Mann–Whitney *U*-test, and categorical values are presented by *n* (%) and tested by chi-squared test or Fisher's exact test.

**Table 4-2:** Outcomes according to histopathology.

	Mature (*n* = 63)	Immature (*n* = 34)	*p*-value
Recurrence	5 (7.9)	9 (26.5)	0.030
Disease free date (months)	126.1 (81.8–183.1)	81.1 (36.5–135.8)	0.007
Additional op	7 (11.1)	13 (38.2)	0.004
Anorectal op	3 (4.8)	2 (5.9)	1.000
Mass excision	4 (6.3)	11 (32.4)	0.002
Perioperative complication	16 (25.4)	25 (73.5)	<0.001
Intraop transfusion	8 (12.7)	22 (64.7)	<0.001
Postop Ventilator	10 (15.9)	20 (58.8)	<0.001
Urinary tract injury	1 (1.6)	2 (5.9)	0.581
Hypoxic damage	0 (0.00)	3 (8.8)	0.075
Postop renal failure	0 (0.00)	2 (5.9)	0.232
Others	0 (0.00)	3 (8.8)	0.075
Late complication	9 (14.3)	7 (20.6)	0.609
Defecation problems	8 (12.7)	1 (2.9)	0.225
Voiding problems	2 (3.2)	6 (17.6)	0.037
ICU stay	10 (6.5–11)	17.5 (9–30)	0.002
Hospital stay	13 (11–15)	18.5 (13–39)	<0.001

Continous values are presented by median (Q1–Q3) and tested by Mann–Whitney *U*-test, and categorical values are presented by *n* (%) and tested by chi-squared test or Fisher's exact test.

## 4 Discussion

Over the decades, prenatal care has advanced, enabling the early detection of fetal malformations, such as SCT, and reducing complications and fetal mortality during delivery. Consequently, surgical resection and long-term complications have become increasingly important considerations. This study aimed to develop a prognostic factor that is relative, objective, easy to calculate, and useful for postnatal counseling.

Both the VWR and LHR were significantly associated with outcomes, including additional operations (e.g., mass re-excision, and bleeding control), perioperative complications, late complications and recurrence. The estimated cut-off values indicated that the larger SCT group, classified based on VWR had higher prevalence of preterm birth, low birth weight, prenatal complications, and prenatal interventions. In groups categorized by the LHR, no significant differences in birth weight or hydrops were observed; however, the larger SCT group included a greater proportion of low-birth-weight, and all hydrops cases occurred in this group. Larger SCT groups for both VWR and LHR also had a higher proportion of immature teratomas. No significant differences were observed in Altman type or tumor rupture; however, higher proportions of intrapelvic tumors (Altman type III and IV) was present in the small-sized group, while ruptured tumors were more frequently observed in the large-sized group for both indicators.

Although all outcomes were significantly associated with VWR and LHR, some subsections in Table 3 did not show significant differences between classified by the cut-off values. For urinary tract injury and hypoxic damage, the small number of cases may have been insufficient for statistical analysis. Additionally, for additional anorectal operations and defecation problems, tumor size did not appear to be a determining factor.

In this study, the cut-off values for each outcome were determined using the maximum Youden index, and the optimal common cut-off value was arbitrarily set by considering the sensitivity and specificity for each outcome. As shown in Table 3, this cut-off value may not fully represent all outcomes, which can be considered a limitation of this study. Furthermore, since each outcome is independent, establishing a single uniform cut-off value may not be feasible, necessitating further multicenter research to determine more precise cut-off values.

Since VWR and LHR are relative size indices, several potential confounding factors related to patient size may exist. As demonstrated in this study, patients with a high VWR were more likely to have a lower body weight, which may be associated with anomalies or underlying lung immaturity, both of which could influence outcomes. However, analyzing the relative size indicators while adjusting confounding factors requires a larger sample size to achieve statistical significance.

In the sub-analysis based on Altman type and histology, no statistically significant association was found between Altman type and outcomes. Several studies have been conducted to determine the relevance between intrapelvic tumor extent defined by the Altman type and recurrence or functional outcomes, but findings remain inconclusive (16–18). Given the relatively small sample size in this study, statistical power may have been limited. Further multicenter studies that adjust for relative tumor size are needed to analyze the relationship between Altman type and prognosis.

In the analysis based on histology, although statistical significance was not observed for several factors, the immature teratoma group generally exhibited poorer outcomes, except for late complications. This finding aligns with previous studies, which have identified histological immaturity as a risk factor for poor outcomes but not for functional impairments (17).

Known risk factors for poor SCT outcomes include early delivery, predominantly solid component tumors, large tumor size, and histological immaturity (17, 19, 20). Rodriguez et al. (15) suggested that the TFR before 24 weeks of gestation is a predictive indicator of fetal outcomes. Their study showed that patients with a TFR greater than 0.12 before 24 weeks of postmenstrual age had an increased risk of complicated pregnancy and perinatal demise due to massive tumor hemorrhage or high output cardiac failure.

Furthermore, Akinkuotu et al. (14) conducted a multi-institutional review and validated TFR > 0.12 as an objective predictor of fetal SCT outcomes, suggesting its utility for prenatal counseling and intervention planning. They also found that fetuses with tumors comprising over 50% solid components had poorer prognoses. This emphasis on tumor morphology aligns with previous studies, which have consistently demonstrated that fetuses with solid tumors experience more adverse outcomes than those with cystic tumors (6, 7, 17, 21). In this study, the impact of the solid tumor portion on prognosis was assessed using volumetry; however, measurement bias arose due to low MRI resolution in small patients and signal differences in cystic portions caused by hemorrhage making automatic calculations difficult. Attempts to estimate the solid mass fraction using biopsy weight were also problematic, as biopsy samples included other soft tissues, making this approach unsuitable. Further preoperative evaluation and calculation techniques need to be developed.

Fetal hydrops and cardiomegaly are also recognized risk factors for poor fetal SCT outcomes, as reported in a recent large cohort study (19). Although fetal hydrops did not show a statistically significant difference in this study, all cases occurred in the large sized SCT group. Regarding cardiomegaly, data were insufficient due to the retrospective nature of this study, preventing meaningful comparison.

In this study, we introduced two indicators —VWR and LHR— that represent tumor volume and length, adjusted for the patient's net birth weight and birth height, respectively. Because all parameters were measured postnatally, these indicators serve as relative measures. The findings suggest that VWR and LHR are predictive factors for prognosis and could be used as triage parameters for patient management. Based on the cut-off values, surgeons may be able to anticipate surgical difficulty and recurrence risk, aiding prognostic counseling. Additionally, VWR and LHR could serve as standard parameters for interdisciplinary collaboration, counseling, and prognostic evaluation across multiple medical departments. Since no significant difference was observed between these two indicators, other centers may select either based on ease of measurement and contextual preference.

SCT studies are characterized by small population sizes, whicn can limit statistical reliability due to the low incidence of the disease. Although this study was constrained by a small patient cohort, it identified an association between SCT relative size and postnatal surgical prognosis. However, the cut-off values varied by outcome, and some estimated values did not correlate with specific outcomes. Further large-scale, multicenter studies are necessary to validate VWR and LHR cut-off values and identify additional prognostic factors affecting surgical outcomes and long-term prognosis.

This study was also limited by the retrospective nature of data collection. There was no analysis of TFR or tumor growth velocity in relation to VWR or LHR due to insufficient prenatal data. Additionally, volumetry was performed using manual tracing, which may have introduced inaccuracies due to poor-quality radiologic images. Other risk factors were not adjusted for, and only relative tumor size indicators were used for outcome comparison, leading to statistical limitations.

The median follow-up period of this study was 1,153 days. Given that recurrences typically occur between 1 and 6 years –postoperatively (17), further research is needed to examine long-term recurrence, complications, and their impact on quality of life. Additionally, further evaluation is required to determine whether VWR and LHR ratio change over time and whether prognosis improves when surgery is performed after these ratios decrease.

## 5 Conclusion

This study demonstrated that VWR and LHR are novel indicators for assessing SCT tumor size in a relative and objective manner. Both indicators were significantly associated with patient prognosis and surgical outcomes. However, due to the limited reliability of the cut-off values, these indicators should be cautiously considered as prognostic parameters for identifying high-risk patients prone to recurrence and complications.

## Data Availability

The original contributions presented in the study are included in the article/Supplementary Material, further inquiries can be directed to the corresponding author.
